# Estimating seed dispersal distance: A comparison of methods using animal movement and plant genetic data on two primate‐dispersed Neotropical plant species

**DOI:** 10.1002/ece3.5422

**Published:** 2019-07-25

**Authors:** Tiziana A. Gelmi‐Candusso, Ronald Bialozyt, Darja Slana, Ricardo Zárate Gómez, Eckhard W. Heymann, Katrin Heer

**Affiliations:** ^1^ Verhaltensökologie & Soziobiologie Deutsches Primatenzentrum – Leibniz‐Institut für Primatenforschung Göttingen Germany; ^2^ Conservation Biology Phillips‐Universität Marburg Marburg Germany; ^3^ Instituto de Investigaciones de la Amazonía Peruana (IIAP) Iquitos Perú; ^4^ Present address: Nordwestdeutsche Forstliche Versuchsanstalt Göttingen Germany

**Keywords:** animal behavior, animal movement, individual‐based modeling, *Leonia cymosa*, *Leontocebus nigrifrons*, parentage analysis, *Parkia panurensis*, *Saguinus mystax*, seed coat, seed dispersal curve, tamarins, zoochory

## Abstract

1. Seed dispersal distance (SDD) critically influences the survival of seedlings, spatial patterns of genetic diversity within plant populations, and gene flow among plant populations. In animal‐dispersed species, foraging behavior and movement patterns determine SDD. Direct observations of seed dispersal events by animals in natural plant populations are mostly constrained by the high mobility and low visibility of seed dispersers. Therefore, diverse alternative methods are used to estimate seed dispersal distance, but direct comparisons of these approaches within the same seed dispersal system are mostly missing.
2. We investigated two plant species with different life history traits, *Leonia cymosa* and *Parkia panurensis*, exclusively dispersed by two tamarin species, *Saguinus mystax* and *Leontocebus nigrifrons*. We compared SDD estimates obtained from direct observations, genetic identification of mother plants from seed coats, parentage analysis of seedlings/saplings, and phenomenological and mechanistic modeling approaches.
3. SDD derived from the different methods ranged between 158 and 201 m for *P. panurensis* and between 178 and 318 m for *L. cymosa*. In *P. panurensis*, the modeling approaches resulted in moderately higher estimates than observations and genotyping of seed coats. In *L. cymosa*, parentage analysis resulted in a lower estimate than all other methods. Overall, SDD estimates for *P. panurensis* (179 ± 16 m; mean ± *SD*) were significantly lower than for *L. cymosa* (266 ± 59 m; mean ± *SD*).
4. Differences among methods were related to processes of the seed dispersal loop integrated by the respective methods (e.g., seed deposition or seedling distribution). We discuss the merits and limitations of each method and highlight the aspects to be considered when comparing SDD derived from different methodologies. Differences among plant species were related to differences in reproductive traits influencing gut passage time and feeding behavior, highlighting the importance of plant traits on animal‐mediated seed dispersal distance.

## 1 INTRODUCTION

Seed dispersal provides the spatial template for subsequent processes that ultimately result in the recruitment of new individuals into plant populations (Nathan & Muller‐Landau, 2000). Seed dispersal impacts seed survival (Connell, 1971; Janzen, 1970; Schupp, Jordano, & Gómez, 2010), determines gene flow within and among populations (Cain, Milligan, & Strand, 2000; He, Lamont, Krauss, & Enright, 2010; Nathan et al., 2008), maintains functional habitat connectivity (Culot, Lazo, Huynen, Poncin, & Heymann, 2010; Lindsell, Lee, Powell, & Gemita, 2015; Ripperger, Kalko, Rodriguez‐Herrera, Mayer, & Tschapka, 2015), and enhances the probability of survival of populations under anthropogenic pressure (Abedi‐Lartey, Dechmann, Wikelski, Scharf, & Fahr, 2016; McConkey et al., 2012; Ruxton & Schaefer, 2012; Snyder, 2011). Measuring the seed dispersal distance (SDD), that is, the distance between the source plant of a seed and its deposition site, is crucial for determining the spatial dimension of the seed shadow and predicting the outcomes of the processes following seed dispersal (Nathan, Klein, Robledo‐Arnuncio, & Revilla, 2012; Nathan & Muller‐Landau, 2000).

Different approaches have been employed to estimate SDD and dispersal distance kernels, each having its specific advantages and limitations (Table 1). Naturally, the method of choice depends on the specific study system and the resources available. Nathan et al. (2012) describe different methods to estimate dispersal distance kernels (i.e., dispersal kernel sensu Nathan & Muller‐Landau, 2000), comparing limitations and uses. However, direct comparisons between methods within the same seed dispersal system are scarce. Mise, Yamazaki, Soga, and Koike (2016) compared SDD estimates for racoon dogs (*Nyctereutes procyonoides*) using the bait‐marker method against the combination of movement data and gut passage, and found comparable results when data were collected from the same region. For the Neotropical legumes *Parkia* spp., coinciding estimates of SSD were obtained by matching the genotypes of seed coats to potential maternal trees and by direct observation of seed dispersal events (Heymann et al., 2012). In the same study area, spatially explicit individual‐based modeling of SDD in *P. panurensis* based on behaviour patterns of the same dispersers provided concordant results (Bialozyt, Flinkerbusch, Niggemann, & Heymann, 2014). Using different methods for determining SDD within the same study system allows evaluating their comparability. This is relevant since often studies that applied different methods need to be compared. In addition, such an evaluation provides valuable information for the decision of which method to choose when direct observations are not possible. For example, when dispersers are difficult or impossible to follow or when several plant individuals of the same species are visited consecutively, and thus, the origin of dispersed seeds cannot be determined. Also, when target plant species of a study system may fail to produce fruits during planned study periods. Such phenological changes may become increasingly frequent as a consequence of global climate change (Abernethy, Bush, Forget, Mendoza, & Morellato, 2018; Cleland, Chuine, Menzel, Mooney, & Schwartz, 2007).

**Table 1:** Comparison of the advantages and disadvantages of different approaches for estimating seed dispersal distance

Method	Data/material required	Advantages	Limitations	References
Observed seed dispersal events (OSD)	Location of feeding events and seed deposition sites. Taxonomic information of local seeds.	Direct method, reduced ambiguity. No expensive equipment needed.	Seed source can only be reliably identified if dispersers do not feed on other fruiting individuals of the same plant species before seed deposition Not applicable to plants with dispersers that are challenging to follow, or for which movement patterns may be altered by human presence.	Yumoto, Kimura, and Nishimura (1999), Stevenson (2000); Wehncke, Hubbell, Foster, Dalling, and Hubbell (2003), Tsuji, Yangozene, and Sakamaki, (2010), Valenta and Fedigan (2010), Heymann et al. (2017)
Genotyping of seed coats (GSC)	Seeds with seed coats still attached. Location of potential source plants and seed deposition sites. Tissue samples from adult plants. Developed genetic markers (e.g., SSRs).	Accurate and reliable identification of source plants. Can be used for accumulation sites and seed traps. No need to follow dispersers nor recognize feeding events when analyzing maternal sources from seed accumulations.	High sampling effort for seeds and adults needed. Finding seeds may become impractical if dispersers have large ranging areas. If multiple dispersers are present , the contribution of each disperser cannot be estimated, except with additional analysis such as bar coding of fecal matter on seeds (González‐Varo et al., 2014).	Dow and Ashley (1996), Godoy and Jordano (2001), Grivet, Smouse, and Sork (2005), Smouse, Sork, Scofield, and Grivet (2012)
Parentage analysis of seedlings (PAS)	Location of potential source plants and seedlings. Tissue samples from adult plants and seedlings. Developed genetic markers (e.g., SSRs).	Measures effective seed dispersal: a more representative measure of the impact seed dispersal will have in the future ecological dynamics of the plant species No need to follow dispersers.	Includes undispersed individuals below the source tree when germination success near source is high. High sampling effort of adults needed. Accurate identification of source plants with nuclear markers only in dioecious plants, in monoecious plants plastid markers are required to discriminate between maternal and paternal sources.	Hamrick and Trapnell (2011), Choo, Juenger, and Simpson (2012)
Phenomenological modeling of SDD based on daily travel path and gut passage time (CMG)	Disperser movement data (Observation, Radio‐tracking, GPS‐tracking) Gut passage time estimate	Estimates distance of all potential seed dispersal events and can be done separately within complex seed dispersal networks to determine disperser‐specific contribution ranges. Can be used to examine differences in seed dispersal patterns between individuals. Can be used to examine the effect of gut passage on seed dispersal patterns. Does not require finding dispersed seeds nor sampling plant tissue. Does not require laboratory access.	Includes movements beyond postfeeding events. Unless dispersers can be observed from feeding to seed deposition in the wild, estimating gut passage times requires capturing or using already captive animals and experimental feeding of fruit species.	Murray (1988), Holbrook and Smith (2000), Westcott et al. (2005), Fuzessy et al. (2017)
Individual‐based modeling of SDD (IBM)	Knowledge of the characteristics of fruiting plant species and disperser behavior. Locations of fruiting plants. Locations of additional fruiting tree species sharing fruiting season	Simulates potential seed dispersal events for single dispersers, even within complex seed dispersal networks. Can be used to analyze the long‐term effect of seed dispersal. Can be used to examine how reproductive traits and resource distribution influence seed disperser movement and seed dispersal patterns. Does not require finding dispersed seeds, sampling plant tissue nor following dispersers.	Parameterization: defining complete descriptions of how dispersers behave in relevant situations. Storage capacity and execution time limitations restrict the number of individual dispersers modeled.	Pakeman (2001), Morales and Carlo (2006), Russo, Portnoy, and Augspurger (2006), Couvreur et al. (2008), Levey, Tewksbury, and Bolker (2008), Uriarte et al. (2011), Bialozyt, Flinkerbusch, et al. (2014)
Seed tracking (not used in this study)	Location of source plants or of experimental seed source and of seed deposition Seed tags (chemical or physical)	Controlled experiment. Seeds can be followed up to include postdispersal events.	Challenging seed retrieval possibly biased by seed trap location when using seed traps. Germination rates are underestimated when seeds are lost. Efficient tracking methods are expensive and time‐consuming	Levey and Sargent (2000), Chauvet, Feer, and Forget (2004), Pons, Pauses, and Pausas (2007), Hirsch, Kays, and Jansen (2012), Jansen et al. (2012), Sork (2016)

Here, we compare different approaches for estimating SDD in two primate‐dispersed plant species. In contrast to many other seed dispersers, primates can be followed for direct observations of seed dispersal events once they are habituated. Thereby, the position of feeding plants and seed dispersal sites can be recorded which allows to obtain the most direct estimate for SDD.

We used data on *Parkia panurensis* (Fabaceae) from previous studies where SDD estimates were based on direct observations of seed dispersal events, maternal identification through genotyping and matching of seed coats, and individual‐based modeling (Bialozyt, Flinkerbusch, et al., 2014; Heymann et al., 2012), and added SDD estimates based on two additional approaches. We replicated these five approaches for *Leonia cymosa* (Violaceae), which has the same seed dispersers as *P. panurensis*, but a different life history. For both species, we compared SDD estimates based on five methods: (a) *o*bserved *s*eed *d*ispersal events (OSD); (b) seed dispersal estimates from maternal identification through *g*enotyping of *s*eed *c*oats (GSC); (c) *p*arentage *a*nalysis of *s*eedlings/saplings (PAS); (d) modeling of SDD through a phenomenological model that *c*ombines *m*ovement data and *g*ut passage times (CMG); and (e) simulating seed dispersal with a mechanistic *i*ndividual‐*b*ased *m*odel (IBM) based on plant distribution data and energy requirements driving dispersers' movement and activity patterns.

**Figure 1:**
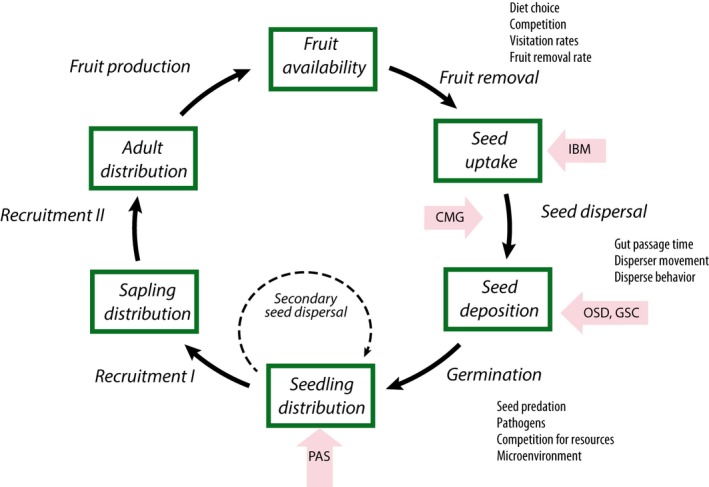
Seed dispersal loop depicting the patterns (boxes) and processes (arrows) that can be measured to assess animal‐mediated seed dispersal and its consequences (modified from Wang & Smith, 2002). Pink arrows indicate the patterns and processes integrated by the different methodologies assessed in our paper to estimate seed dispersal distances: observed seed dispersal events (OSD), genotyped seed coats (GSC), parental analysis of seedlings/saplings (PAS), combination of movement data and gut passage (CMG), and individual‐based modeling (IBM)

Each of these methods integrates over different processes of the “seed dispersal loop” described by Wang and Smith (2002); thus, we expect differences among the methods depending on the processes they integrate (Figure 1). Observations of seed dispersal events (OSD) and genotyping of seed coats (GSC) measure the distance between maternal tree and seed deposition and thereby provide the most direct measures of SDD. By integrating across several processes of the loop, namely fruit production, fruit removal, and seed dispersal, the estimates obtained from OSD and GSC are crucially influenced by the foraging behavior of the animals. In contrast, parentage analysis (PAS) is based on seedling distribution, and thus, integrates also postdispersal processes such as germination success and secondary seed dispersal. Thereby, PAS considers effective seed dispersal, as opposed to realized seed dispersal (Schupp et al., 2010). The phenomenological model (CMG) uses recorded movement patterns and gut passage time estimates, and thus, the SDD estimate is based on processes taking place before the actual seed deposition. Finally, the individual‐based model (IBM) simulates seed deposition based on information of resource distribution and fruit consumption following energetic demands of the seed dispersers; and thus, the estimate integrates fruit availability and seed uptake.

By applying these methods to two plant species with the same exclusive seed dispersers, we can further assess different methodological approaches in the context of plant life history traits and their impact on foraging behavior.

## 2 MATERIALS AND METHODS

### 2.1 Study site

We collected data at the Estación Biológica Quebrada Blanco (EBQB), located at 4°21′S, 73°09′W in northeastern Peruvian Amazonia. For details of the study area, see Heymann (1995) and Culot et al. (2010).

### 2.2 Plant species

We used data from two different plant species, *Leonia cymosa* (Violaceae) and *Parkia panurensis* (Fabaceae). *Leonia cymosa* is an understory tree and has a population density of five and 11 adults per hectare within the home ranges of two tamarin study groups at EBQB, group 1 and group 2, respectively. It produces berries with two to seven seeds embedded in a fibrous pulp. Fruits ripen asynchronously within and between trees throughout several weeks, for up to three months. During weekly counts, 25–125 fruits can be present per tree (Reinehr, 2010). Feeding visits by tamarins vary between <1 and 10 min (1.9 ± 0.1 min.; mean ± *SD*), with generally only one or two (maximum five) tamarins feeding in a single tree (Reinehr, 2010). On average, tamarins visit 7.5 trees per day (range: 1–14 trees) and generally feed on several *L. cymosa* trees consecutively (Reinehr, 2010). This “trap‐lining” feeding strategy (Garber, 1988) makes it challenging to assign seeds to a source tree during the observation of seed dispersal events.

*Parkia panurensis* is a canopy tree and occurs at a population density of around one adult per hectare at EBQB. It produces pods with 15–25 seeds surrounded by an edible sticky gum (Hopkins, 1986). Within a single tree, ripe pods may be present for up to 12 weeks and within the population for up to four months. Feeding visits of tamarins' range between three and 46 min (11.8 ± 0.7 min.; mean ± *SD*), depending on the number of ripe pods available at a given moment, and generally, all group members feed simultaneously on fruits of a single tree (E. W. Heymann, pers. obs.; Feldmann, 2000). Tamarins may visit the same tree up to four times per day, without a trap‐lining feeding strategy. Therefore, seeds can very often be reliably assigned to a single source tree (Heymann et al., 2012).

### 2.3 Seed dispersers

At EBQB, the seeds of both plant species are exclusively dispersed by two species of tamarin monkeys, *Saguinus mystax* and *Leontocebus nigrifrons* (Callitrichidae). This has been confirmed through focal tree observations and camera trapping (Heymann et al., 2012; Reinehr, 2010). These primates live in groups of three to 12 individuals and form mixed‐species troops in which members from both species move through a shared home range in a highly coordinated way (Heymann & Buchanan‐Smith, 2000). Home‐range size of tamarins at EBQB varies between c. 25 and 50 ha, and mean daily path length (i.e., the length of the route travelled from sleeping site to sleeping site) varies between 600 and 3,000 m (mean: 1,700 m; Smith, 1997). Daily travel paths are not linear but have variable shapes (e.g., concentric, meandering; e.g., Figure S2).

### 2.4 Collection of observational data

Tamarin groups at EBQB are well habituated to the presence of human observers and can be observed at close range. For this study, we used two observational datasets. First, we derived data on feeding and seed deposition events of *L. cymosa* and *P. panurensis* from studies by Knogge (1999), Culot (2009), and Heymann et al. (2012). These included location and time of feeding and defecation by both tamarin species. Defecations containing one or more seeds were defined as seed dispersal events (Knogge & Heymann, 2003). We only considered events where no other *L. cymosa* or *P. panurensis* were consumed between feeding and seed deposition. Second, we used movement data of tamarins sampled independently from the seed dispersal studies cited above. Observations were conducted for a total of 62 days, with a mean of 7.7 days per month (*SD*: 2.8 days) from December 2012 to July 2013. Positional data were recorded every 30 min. Before GPS devices were available, positions were determined in reference to the 100 m × 100 m trail grid at EBQB and in reference to previously marked and mapped trees in the study by Knogge (1999). Thereafter, with a Garmin GPSMap 76CSx using the Universal Transverse Mercator (UTM) projection .

### 2.5 Sampling of plant material

**Figure 2:**
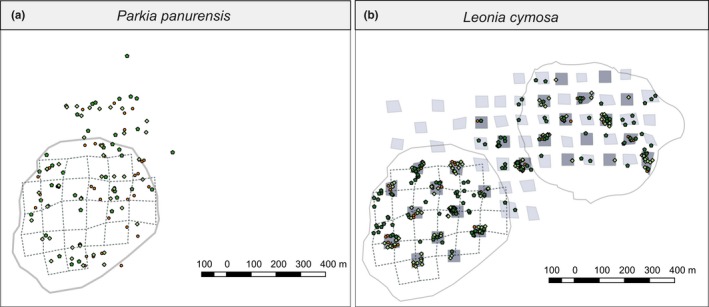
Sampling map for *Parkia panurensis* (a) and *Leonia cymosa* (b). Maps show locations of sampled seedlings ◊, saplings ○, and adult trees ⌂. Home ranges of tamarin group 1 and group 2 are depicted by solid gray lines, trails within group 1 are depicted by dashed lines, as a reference. Leaf tissue sampling for *P. panurensis* was limited to the home range of tamarin group 1. Leaf tissue sampling for *L. cymosa* was extended across home range areas of group 1 (left, ha. 38.9) and group 2 (right, ha. 38.1), and across sampling years (2014–2015). Quadrats depicted in dark gray were sampled in 2014 and those depicted in light gray in 2015. Additional adults were sampled in transects following the trail system in group 1

We collected leaf samples from 467 potential offspring (seedlings and saplings ≤ 250 cm and leaf number < 50) and 194 potential parents (adults > 250 cm) of *L. cymosa* in 25 quadrats of 50 m × 50 m within the home range of tamarin group 1 (12 quadrats, corresponding to c. 15% of the home‐range area) and group 2 (13 quadrats, corresponding to c. 15% of the home‐range area). Quadrats were located at the crossings of the trail system that spans the study site (Figure 2b). To increase the number of candidate parents, we additionally sampled along 53 transects of 15 m × 100 m in group 1 and additional quadrats on remaining path intersections in group 2. For storage, we either dried the leaf samples on silica gel beads or soaked Whatman FTA PlantSaver cards (GE Healthcare Lifesciences) with smashed leaf material (see Appendix S1). When *L. cymosa* trees were fruiting in 2016 (no fruiting took place in 2014 and 2015), we collected seeds of *L. cymosa* from droppings excreted by tamarins during focal observations, recorded the location of seed deposition, and stored the seeds in a saturated saline solution.

For *P. panurensis*, we used genetic data from plant material of 85 potential offspring (seedlings and sapling, height > 1.3 m and diameter at breast height (dbh) <20 cm), 33 potential parents (adults, dbh > 20 cm), and 92 seeds, sampled for Bialozyt, Luettmann, et al. (2014). For this study, a full inventory of adult *P. panurensis* was carried out within the home range of tamarin group 1, while seedlings and saplings were sampled by randomly overlaying a 50 m × 50 m grid over a map of the home range of tamarin group 1 (Figure 2a). Intersections of this grid were taken as central points for 50 m × 50 m quadrants where seedlings and saplings were sampled exhaustively. Geographic coordinates of all sampled individuals were recorded with a Garmin GPSMap 76CSx.

### 2.6 Genotyping with microsatellite markers

To prepare the leaf samples of seedlings, saplings, and adult trees for DNA extraction, we ground the leaves using a Retsch mill (Haan, Germany). To prepare seed coat samples, we rehydrated seeds at room temperature and then separated seed coats carefully from the embryo and ground them in the Retsch mill. DNA was extracted from ground leaves and seed coats following the modified CTAB protocol with ATMAB (Dumolin, Demesure, & Petit, 1995). For *L. cymosa*, DNA of each sample was genotyped using eleven nuclear microsatellites, following the protocol described in the Appendix S1. For *P. panurensis*, we used genotype information from nine nuclear microsatellite markers from Heymann et al., (2012).

### 2.7 Data analysis for estimating seed dispersal distance

#### 2.7.1 Observed seed dispersal events (OSD)

For each observed dispersal event, we determined the SDD as the linear distance (i.e., Euclidean distance) between the location of feeding and defecation.

#### 2.7.2 Maternal identification from genotyping of seed coats (GSC)

The seed coat is of maternal origin, and thus, a precise match between the genotype of a seed coat and the genotype of an adult identifies the proper mother. We matched genotypes from seed coats to adult genotypes using GenAlex v. 6.501 with no mismatches allowed (Peakall & Smouse, 2006). To estimate SDD, we determined the linear distance between the source tree and the dispersed seed based on the recorded UTM coordinates.

#### 2.7.3 Parentage analysis of seedlings and saplings (PAS)

We used the CERVUS software v3.0.7 (Slate, Marshall, & Pemberton, 2000) to determine potential parents for the genotyped seedlings/saplings in our study area. We selected strict confidence intervals (95%) for the parental analyses using maximum likelihood framework. We ran the preliminary simulation with the following parameter settings: number of candidate parents was set to 194 for *L. cymosa* and to 33 for *P. panurensis*, proportion sampled was set to 0.15 for *L. cymosa* based on sampling areas in relation to the total home‐range area and to 0.99 for *P. panurensis* where we are confident that all adult trees within the tamarins' home range were sampled. We determined the genotyping error as 0.01 using GenAlex, and we only included individuals with a minimum of six typed loci for *L. cymosa* and five for *P. panurensis*. Finally, we determined the linear distance between the resulting parents and offspring as the estimate for SDD based on the recorded UTM coordinates.

Given that maternal sources of seedlings and saplings were unknown in *L. cymosa* and *P. panurensis*, we assumed that both parents could be either mother or father to avoid potential bias. Following this assumption, we used all possible parent–offspring combinations to calculate a mean SDD and density distance kernels. As observations of seed dispersal events showed that SDD by tamarins did not exceed 709 m (*n* = 1,884; Knogge, 1999), which corresponds to the diameter of a tamarin home range. We excluded parent–offspring pairs with distances beyond 700 m from this analysis. Since 700 m is the maximum diameter of tamarin home ranges, parent–offspring relationships beyond this distance are most likely caused by pollination rather than seed dispersal.

#### 2.7.4 Phenomenological model combining movement data with gut passage time (CMG)

**Figure 3:**
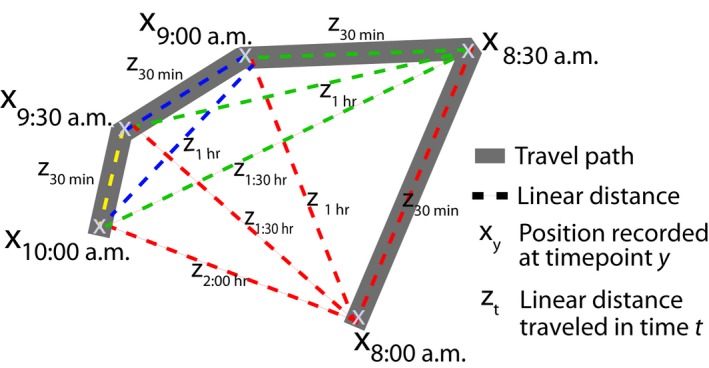
Graphical example of the procedure to estimate seed dispersal distance using a *c*ombination of *m*ovement data with *g*ut passage times (CMG). To obtain a series of linear distances (dashed lines), we calculated the linear distances between scan points (X) that were recorded every 30 min throughout the day along the travel path of the tamarins. This way, we obtained a series of distances for different time intervals from 30 min up to 9 hr for each day following the tamarins' daily activity pattern

To model SDD based on movement data of seed dispersers and gut passage time, we modified the approach used by Murray (1988). As Murray (1988), we calculated linear distances between scan points for each daily travel path, considering the time interval between each pair of scan points (Figure 3). For this purpose, we used the movement data recorded in 2013 (see Section 2.4) restricted to the respective fruiting seasons of the two species during that sampling year to account for monthly variation in travel path length (*L. cymosa*: March–May, *n* = 31 days; *P. panurensis* May–July, *n* = 31 days).

From this movement data, we derived linear distances with our custom R function *linear.distances*() (see Appendix S2). In contrast to Murray's approach, we did not limit the analysis to scan points after visits to food plants. Thus, our method is also applicable under conditions where no information on the timing of feeding is available, for example in cases where animals are tracked remotely.

Subsequently, we only considered linear distances within the time interval of gut passage for the seeds of each species. We estimated gut passage time as the time lag between feeding and defecation based on data collected by Knogge (1999) and Culot (2009). Since resting times can increase gut passage time without increasing SDD, we used a conservative range and included gut passage times within the 5% confidence interval (CI) as lower limit and the 80% CI as upper limit. CIs were calculated with the *npquantile*() function from the *np* package (Racine & Hayfield, 2018). For *P. panurensis*, the resulting gut passage time estimate was 30–240 min (*n* = 196).* For L. cymosa,* we only had few observation datapoints available and thus used the minimum and maximum value observed, resulting in a gut passage time estimate of 120–240 min (*n* = 3).

#### 2.7.5 Individual‐based modeling of seed dispersal events (IBM)

In the individual‐based model (IBM), we simulated tamarin movement and feeding activity in order to maintain homeostasis and, as a result, tamarins dispersed seeds after a predefined gut passage time, following Bialozyt, Flinkerbusch, et al. (2014). The model was originally developed for *P. panurensis* based on data from tamarin group 1. Only *P. panurensis* trees on which feeding events were observed on site during the 2008 observation period were considered. Furthermore, for the purpose of the simulation it was assumed that there were no other species used for feeding. This assumption was valid for these simulations because the data collection had been carried out during a time span when *P. panurensis* was nearly the exclusive fruit source for the tamarins.

For *L. cymosa*, we adjusted the previous model in four critical aspects. First, since *L. cymosa* is never the only fruit source available in this area, we needed to add other species as fruit source to allow for enough energy input during the daily routine of the tamarins. We used the other species of feeding trees observed during *L. cymosa'*s fruiting season in 2013 as additional fruit sources. Furthermore, not all *L. cymosa* trees fruit yearly; therefore, we used the subset of *L. cymosa trees* (*n* = 8) observed that same year. Second, *L. cymosa* contains 415–642 mg of soluble sugars per gram of dry matter, whereas *P. panurensis* contains 811 mg/g (Peres, 2010; Pfrommer, 2009). Therefore, we adjusted the mean energy level provided by the trees in the simulation model (Table S4). Third, different time intervals in feeding trees for a single feeding event were implemented to reflect the respective fruit crop size and the resulting shorter feeding times in *L. cymosa*. Fourth, we adjusted gut passage time for *L. cymosa* according to field observations of seed dispersal events reported by Knogge (1999) and Culot (2009). All other parameters were kept at values of the previous *P. panurensis* simulation (Bialozyt, Flinkerbusch, et al., 2014). We simulated daily movements for a total of 200 days, from which we obtained seed deposition and maternal location as an output in UTM. We then determined the linear distance between dispersed seeds and maternal trees.

### 2.8 Statistical analysis

We estimated mean SDD values for each method by bootstrapping distance values (*n* = 10,000 resamplings) using the *boot_mean*() function from the “boot” package in R (Canty & Ripley, 2017; Davison & Hinkley, 1997). We evaluated differences between methods and species with the nonparametric Kruskal–Wallis test using the *kruskal.test*() function from the “stats” package in R (R Core Team, 2018). We did further post hoc comparisons with the nonparametric multiple comparison test and Bonferroni corrections using the *pairwise.wilcox.test*() function from the “stats” package in R (R Core Team, 2018).

To estimate dispersal kernels, we determined the empirical frequency distribution (i.e., density distance kernels) of dispersal distances for each method by adjusting a nonparametric function (smooth spline curve) and its confidence envelope estimated by bootstrapping (*n* = 100 resamplings) using the *mykernel*() function (Jordano, 2016). Bandwidth size was calculated with the function *density*() from the “stats” package (R Core Team, 2018).

Finally, to compare dispersal kernels between methods, we estimated the probability distribution of all methods using the *stat_ecdf*() function from the “ggplot2” package in R (Wickham, 2016). Subsequently, we tested differences between the empirical cumulative distribution functions of each method with the two‐sample Kolmogorov–Smirnov test, which is sensitive to differences in both location and shape of the cumulative distribution function. For the Kolmogorov–Smirnov test, we used the *ks.test*() function from the “stats” package in R (R Core Team, 2018).

## 3 RESULTS

### 3.1 Comparison among methods

**Figure 4:**
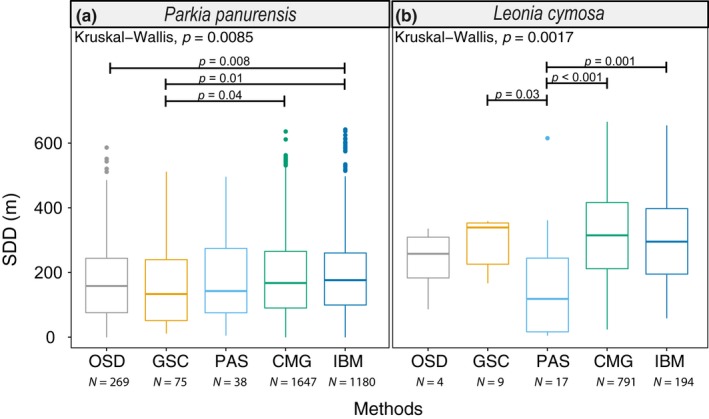
Seed dispersal distance estimates based on the five methods used in this study: observed seed dispersal events (OSD), genotyped seed coats (GSC), parental analysis of seedlings (PAS), combination of movement data and gut passage (CMG), and individual‐based modeling (IBM) for (a) *Parkia panurensis* and (b) *Leonia cymosa*. Horizontal lines represent medians, boxes show the 25%–75% quartiles, and dots are outliers. Bars above the boxplots indicate differences among methods based on a Kruskal–Wallis test and multiple pairwise comparisons with Wilcoxon rank sum test

Depending on the method used, mean SDD estimates ranged between 158 and 201 m for *P. panurensis* and between 178 and 318 m for *L. cymosa *(Table S5). Overall, methods varied significantly in the resulting SDD estimates for each species (*P. panurensis*: H_(4)_ = 13.7, *p* = 0.009; *L. cymosa*: H_(4)_ = 17.3, *p* = 0.002). Specifically, Wilcoxon pairwise comparisons revealed that in *P. panurensis*, SDD estimates from individual‐based modeling (IBM) were significantly higher than those from observations (OSD) and maternal identification of seed coats (GSC), and SDD estimates from the phenomenological model (CMG) were significantly higher than those from GSC (Figure 4a). In *L. cymosa*, instead, SDD estimates from parentage analysis of seedlings/saplings (PAS) were significantly lower than those from GSC, CMG, and IBM (Figure 4b).

**Figure 5:**
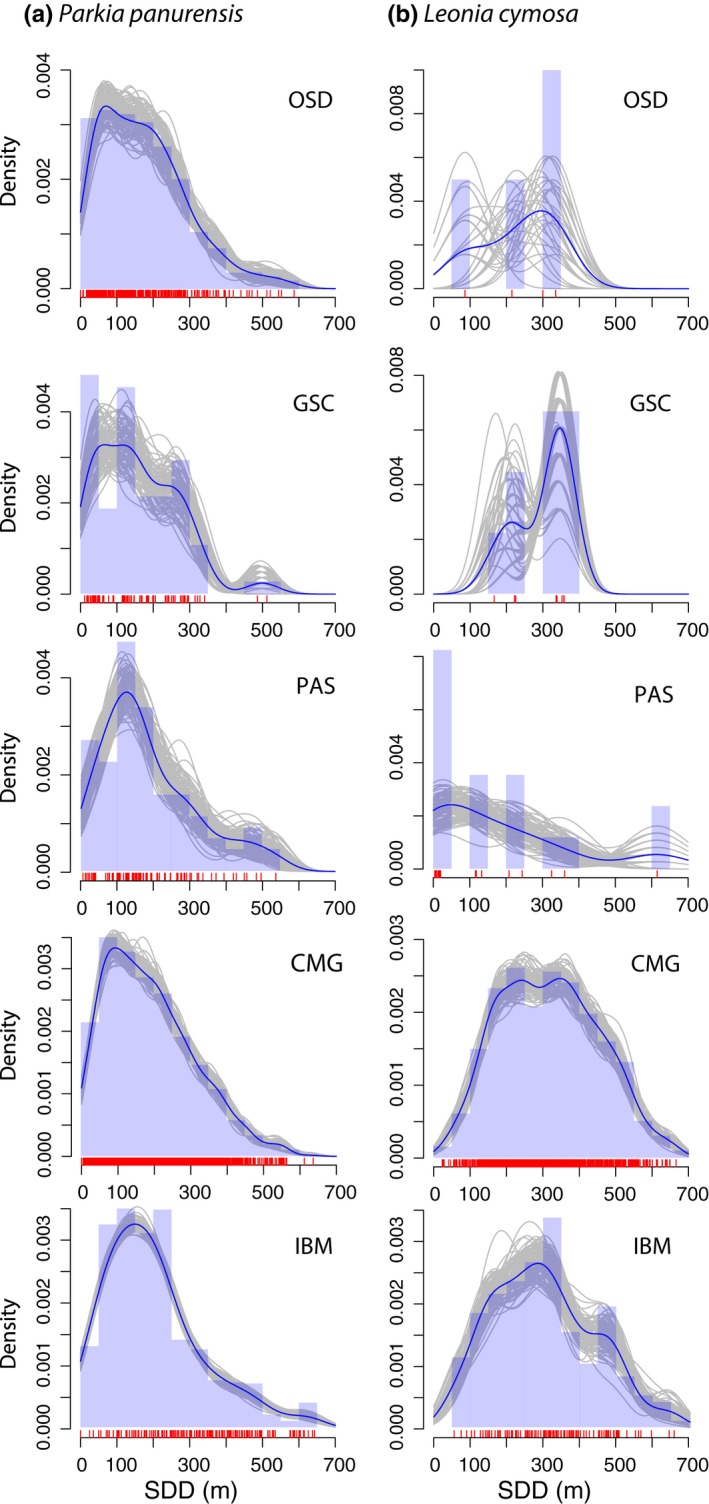
Distribution of seed dispersal distances for the five methods used for *Parkia panurensis* (a) and *Leonia cymosa* (b). The figures show, for each method, the density of dispersal events within the distance class (blue bars), a nonparametric smoothing spline fit to the empirical distance distributions (blue lines), together with bootstrapped estimates (gray lines). Red vertical bars along the *x*‐axis represent each observed dispersal event. Abbreviations refer to the applied methods to estimate SDD: observed seed dispersal events (OSD), genotyped seed coats (GSC), parental analysis of seedlings/saplings (PAS), combination of movement data and gut passage (CMG), and individual‐based modeling (IBM).

For *P. panurensis*, IBM was the only method producing a significantly different dispersal kernel (Kolmogorov–Smirnov test: IBM vs. OSD: *p* = 0.02; IBM vs. GSC: *p* = 0.03; IBM vs. CMG: *p* = 0.002; Figure 5a). All methods except for IBM produced significantly right‐skewed dispersal kernels, that is, curves with an extended tail to the right. Furthermore, in *P. panurensis*, the cumulative SDD curves derived from all methods converged at low distances (Figure S3a). In *L. cymosa*, instead, the shape of the cumulative SDD curves were more variable (Figure S3b), and only the dispersal kernel derived from PAS was significantly less right‐skewed than those from GSC, CMG, and IBM (Kolmogorov–Smirnov test, PAS vs. GSC: *p* = 0.03, PAS vs. CMG: *p* < 0.001, and PAS vs. IBM: *p* < 0.001, Figure 5b).

### 3.2 Comparison between species

SDD estimates were significantly higher in *L. cymosa* (266 ± 59 m; mean ± *SD*) than in *P. panurensis* (179 ± 16 m; mean ± *SD*; Kruskal–Wallis, H_(4)_ = 557.5, *p* < 0.001, Table S5). The difference was consistent among methods except for OSD and PAS (Wilcoxon, OSD: *p* = n.s.; GSC: *p* < 0.001; PAS: *p* = n.s.; CMG: *p* < 0.001, IBM: *p* < 0.001).

## 4 DISCUSSION

Our analyses revealed that different methods resulted in statistically different SDD estimates. However, differences between methods were diversely expressed in the two tree species examined here. Specifically, modeling methods showed longer SDD estimates in *P. panurensis*, while parentage analysis resulted in lower SDD estimates in *L. cymosa*. Irrespective of these differences, estimated SDD were significantly higher for *L. cymosa* than for *P. panurensis*.

Here, we first discuss how the intraspecific differences among methods are related to species‐specific plant traits. Subsequently, we assess more generally the merits and limitations of each methodology. Finally, we discuss the reasons behind interspecific differences in SDD estimates between the two plant species.

### 4.1 Intraspecific methodological differences in estimated seed dispersal distance

For *P. panurensi*s, we observed the typical right‐skewed distribution of seed dispersal (Clark, Silman, Kern, Macklin, & HilleRisLambers, 1999; Nathan et al., 2008) independently of the method used. However, mean SDD estimates were higher with modeling methods (CMG and IBM) than with the other methodologies. Modeling methods such as these do not account for real seed deposition events (Figure 1), likely missing defining disperser behavior between seed uptake and seed deposition that might be related to the species‐specific feeding event or to other resource trees surrounding the feeding tree. For example, by combining daily travel paths with gut passage estimates, the phenomenological model (CMG) does not account for the fact that tamarins frequently rest nearby after feeding in the large fruit crops of *P. panurensis* (Knogge, 1999). Individual‐based modeling (IBM), instead, does account for fruit crop size and foraging decisions influenced by energetic needs, but the modeling outcome is affected by the spatial distribution of adult plants and plant population density (Pegman, Perry, & Clout, 2017). Low adult population density in *P. panurensis* might lead to an overestimation of SDD, especially when not accounting for the presence of other fruit sources in the study area. However, the magnitude of the observed differences between modeling methods (CMG, IBM) and reference methods (OSD, GSC) were relatively small (188 and 201 m vs. 158 and 172 m, respectively) particularly when taking the measurement error of GPS devices into consideration. Therefore, none of the methods provided a gross over‐ or underestimation of the real SDD despite statistical significance.

In *L. cymosa*, parentage analysis of seedlings/saplings (PAS) resulted in significantly shorter SDD estimates than all other methods (178 m vs. 234–318 m, Table S5) with a right‐skewed dispersal kernel while SDD estimates from other methods were normally distributed. Even though we cannot exclude the possibility that the small sample size for OSD and GSC plays a role in this statistical difference with PAS, PAS can underestimate SDD if nondispersed germinated seedlings are included in the sampling. Lower values of SDD derived from PAS are in line with field observations that showed a high percentage of fruits and seeds are discarded by the tamarins below the fruiting trees (28%–38%, Feldmann, 2000; 40%, Reinehr, 2010). Tamarins also accumulate seeds of both species under sleeping sites (Knogge, 1999; Culot, 2009); however, we did not observe lower SDD estimated by PAS in *P. panurensis*. This can be attributed either to sampling bias and differences in population density (see Section 4.2), or to differences in density‐dependent seed survival and seedling germination. For *L. cymosa*, we found seedlings very close to each other, including a few clusters (Figure 2). In contrast, the survival rate of seeds of *P. panurensis* beneath fruiting trees is very low (2%, Feldmann, 2000), suggesting a high density‐dependent mortality.

Overall, our results revealed differences in SDD estimates among methods that are likely related to the different processes of the seed dispersal loop each method integrates and to differences between plant species within each process.

### 4.2 Methodological assessment

Among the methods used to estimate SDD, seed dispersal observations (OSD) and genetic identification of maternal source from seed coats (GSC) provide the most reliable information and we regard them as references for SDD estimation. However, using OSD and GSC is not always feasible (Table 1), for example, when the source plant is not easily determined by observations or when seeds and fecal samples cannot be easily collected. Our results showed that estimates obtained through phenomenological (CGM) and mechanistic (IBM) modeling resulted in similar or, at most, slightly higher SDD estimates compared with the reference methods (OSD and GSC). However, these two methods are very sensitive to the input data (Table 1). Specifically, IBM is sensitive toward the spatial distribution of fruiting plants (Pegman et al., 2017) and detailed knowledge of disperser behavior and of plant life history would allow for a more accurate definition of input parameters. In the case of CMG, accuracy of estimates should increase with a higher temporal resolution of movement data. The rapid development of GPS technology, with smaller and more accurate tracking devices (Abedi‐Lartey et al., 2016; McMahon et al., 2017; Oleksy, Giuggioli, McKetterick, Racey, & Jones, 2017; Sánchez‐Giraldo & Daza, 2019) for small to medium‐sized seed dispersers will increase the precision and accuracy of seed dispersal estimates based on CMG. However, these will only be of use if reliable data of gut passage time are available, which are crucial for the CMG method as well. In both tamarin species, gut passage times show considerable variation within and between plant species (Knogge, 1999). Nonetheless, field observations of gut passage time are also not feasible when the maternal source is not easily identified. An alternative to field observations of gut passage times are estimates derived from captive animals (Abedi‐Lartey et al., 2016; Holbrook, 2011; Holbrook & Smith, 2000; Westcott, Bentrupperbäumer, Bradford, & McKeown, 2005). However, it remains to be determined how representative results from captive animals are, as restriction of movements affects gut motility (Holdstock, Misiewicz, Smith, & Rowlands, 1970; Oettlé, 1991). For example, gut passage time of seeds increased by up to 80% with physical activity in mallard ducks (Kleyheeg, van Leeuwen, Morison, Nolet, & Soons, 2015).

As mentioned above, PAS is the only method based on seedling distribution and thus provides an estimate for effectively dispersed seeds (Schupp, 1993) rather than of realized seed dispersal as OSD and GSC. This is more representative of the impact seed dispersal will have in the future ecological dynamics of the plant species, but is also an important difference to consider when pooling and comparing data from different methods. In addition, PAS‐based estimates strongly depend on whether seedlings below adult trees are integrated or not within the sampling scheme. These seedlings might originate either from short‐distance seed dispersal or from undispersed seeds (Sezen, Chazdon, & Holsinger, 2009). Empirical data would be needed to determine the number of short‐distance dispersal events and the degree of density‐dependent mortality below adult trees. Such data could, for example, originate from a complementary use of PAS and genotyping of seed coats (GSC) that can be used to evaluate postdispersal processes and germination success (e.g., Augspurger, 1983; González‐Martínez, Ersoz, Brown, Wheeler, & Neale, 2006; Swamy et al., 2011; Bontemps, Klein, & Oddou‐Muratorio, 2013). In our study, the higher population density of *L. cymosa* in combination with the quadrat sampling scheme might lead to a higher proportion of undispersed seedlings sampled. This provides an alternative explanation as to why PAS resulted in lower SDD estimate and stronger right‐skewedness than other approaches in *L. cymosa*, while this was not the case for *P. panurensis*.

Long‐distance seed dispersal (LDD) events have a strong impact on plant community composition (Cain et al., 2000), and methodologies assessed in our study might also differ in their ability to provide reliable estimates of such events. LDD is often associated with unusual behavior of the disperser (Nathan et al., 2008), and in our study system, LDD would happen in the extremely rare events when single tamarin individuals leave their group while transporting the respective seeds in their guts. Sampling of behavioral data and of plant individuals for this study was confined to the home‐range areas of the tamarin groups. Therefore, further research is needed to assess methodological differences regarding LDD.

### 4.3 Interspecific differences in seed dispersal distance

Despite differences in SDD estimates among the compared methods, estimates were consistently lower for *P. panurensis* than for *L. cymosa*. These differences can be explained by differences in reproductive traits, that is, fruit characteristics and fruit crop size, and population density. First, pods of *P. panurensis* contain seeds covered by a gelatinous exudate, while *L. cymosa* seeds are covered by a fibrous pulp firmly attached to the seeds. Knogge (1999) showed that gut passage times for seeds with fibrous pulp are longer than for seeds with gelatinous pulp, and generally, longer gut passage times result in longer SDD (Fuzessy, Janson, & Silveira, 2017). Second, movement patterns and foraging behavior of tamarins differ when feeding in *L. cymosa* and *P. panurensis*. In *L. cymosa*, fruit crop sizes are small and population density high; therefore, only a few individuals feed simultaneously in the same tree, and the same tree is rarely revisited on a given day (Reinehr, 2010). In contrast, the entire group feeds simultaneously on the large fruit crop of single fruiting *P. panurensis* individual, and often the same tree is repeatedly visited on the same day, which potentially shortens SDD (E. W. Heymann, unpubl. data). Overall, our results are in line with what we can derive from the set of life history traits in the two species. However, to obtain more general conclusions regarding the relationship of plant traits, foraging behavior, and resulting SDD, further research could either implement different reproductive traits into the IBM and analyze SDD outcomes or compare SDD estimates from a greater number plant species with different sets of life history traits.

## 5 CONCLUSIONS

By comparing different methods for estimating SDD in a single seed disperser system, our study allows identifying the merits and limitations of each method. Despite significant differences, the modeling approaches and parentage analysis provided estimates without biologically relevant deviations from the reference methods (OSD and GSC). Our study can serve as a guideline for evaluating and comparing studies that employed diverse approaches to estimate SDD, in particular when comparing among methods that measure realized versus effective seed dispersal.

Further, the difference in SDD between the two studied plant species highlights the importance of plant traits in the foraging behavior of animal seed dispersers. Thus, future studies should strongly consider that animal‐mediated dispersal kernels are dependent on parameters such as plant density, reproductive traits, and species‐specific gut passage times, in particular when extrapolating SDD from different plant species.

## CONFLICT OF INTEREST

None declared.

## AUTHOR CONTRIBUTION

TG, EWH, and KH conceived and designed the analysis. TG and RZ collected plant material, and DS collected animal movement data. TG performed the genetic analysis, the *OSD*, *GSC*, *PAS*, and *CMG* analysis, and the statistical analysis and prepared the figures. RB performed the *IBM* analysis. TG, EWH, and KH wrote the manuscript, and TG, DS, RZ, RB, EWH, and KH revised the final version of the manuscript.

## Data Availability

All datasets and the codes for the CMG and IBM method are available at the repository online, Zenodo (genotype dataset for *L. cymosa*, record: 1470486#.XOxlFogzZPY CMG, record: 1470486#.XOxlFogzZPY; IBM, record: 1471479#.XOxk_4gzZPY, and the R code for the CMG method is also available in the Appendix.
