# Supplementary material for: Estimating seed dispersal distance: A comparison of methods using animal movement and plant genetic data on two primate‐dispersed Neotropical plant species
Source: Ecol Evol. 2019 Jul 25;9(16):8965–77. doi: 10.1002/ece3.5422 (PMC6706201; doi:10.1002/ece3.5422)
Supplement: Supplementary file 4 [file ECE3-9-8965-s004.docx]

**Table S1** Description of specimen locations from sampled individuals

| Species | Voucher specimens | Collection locality | Geographic coordinates |
| --- | --- | --- | --- |
| *L. cymosa* | TA Gelmi-Candusso 025022 EBQB (AMAZ) | Estacion Biologica Quebrada Blanco (EBQB), Iquitos, Peru | 4° 21’ S,  73° 09’ W |
| *L. cymosa* | R Zarate 20272 AM (HH) | Alpahuayo-Mishana (AM), Iquitos, Peru | 4° 29' S,  73° 35' W |
| *L. crassa* | R Zarate 20273 AM (HH) | Alpahuayo-Mishana (AM), Iquitos, Peru | 4° 29' S,  73° 35' W |
| *L. glycicarpa* | R Zarate 20271 AM (HH) | Alpahuayo-Mishana (AM), Iquitos, Peru | 4° 29' S,  73° 35' W |
