# Supplementary material for: Estimating seed dispersal distance: A comparison of methods using animal movement and plant genetic data on two primate‐dispersed Neotropical plant species
Source: Ecol Evol. 2019 Jul 25;9(16):8965–77. doi: 10.1002/ece3.5422 (PMC6706201; doi:10.1002/ece3.5422)
Supplement: Supplementary file 5 [file ECE3-9-8965-s005.docx]

**Table S2** Description of genetic markers for each microsatellite loci, annealing temperature (Ta) and Genbank accession numbers.

| Primer | Primer sequences (5’- 3’) | Repeat motif | Size (bp) | Ta (°C) | GenBank accession no. |
| --- | --- | --- | --- | --- | --- |
| Leo80 | F: TTTAGCGGTACGCTTTTCAC  R: AAAAGCATGGCCTTTCCAGC | (TTGT)7 | 224-236 | 53 | MF002374 |
| Leo89 | F: GTTCGCCTCACCATAAAGGC  R: AAGAGTGAGCATGCGTGAAG | (TTTC)8 | 197-221 | 55 | MF002375 |
| Leo94 | F: AAACCCTTGTTTTCGAATTTAGATG  R: GGGGCCAATTTGACTTTTTGC | (TTTG)8 | 220-236 | 59 | MF002376 |
| Leo270 | F: GTACTTGCACCATGCCACC  R: TAGCACTTCTGCACTTGTTG | (AAAC)8 | 110-122 | 55 | MF002377 |
| Leo466 | F: AGCATAGACACCACGGCTAC  R: AACTTGATCCCCAGTTTGGC | (AAGA)9 | 196-216 | 55 | MF002378 |
| Leo1842 | F: ACCCCATGACCCTTTAGTGC  R: TTTTATGTTAAGTTCTTGCAATGGG | (AAAG)7 | 224-244 | 59 | MF002379 |
| Leo2254 | F: ATGCACCATTGAACTTGGTC  R: AACCCACGCCTTTTATGCAG | (AAGA)8 | 126-166 | 53 | MF002380 |
| Leo2428 | F: TTATATTTGTCCTCCCTTCTGATAAC  R: GATCAATGGCTGCTCTCGTG | (TTTG)7 | 100-112 | 59 | MF002381 |
| Leo2433 | F: AGGAGTTAGCAATACAAAGTGAGTG  R: TCGTGTTAATCCCTTCTTTCCC | (ATAC)14 | 216-268 | 59 | MF002382 |
| Leo2833 | F: ACTATGTCACCTCACAAGCC  R: CTGAAATGCACCCTACGGAAC | (CATA)8 | 178-206 | 53 | MF002383 |
| Leo2853 | F: TTGCAAGGCACAATGACGAC  R: TACACAGTGCCAACATGCAG | (ATAC)8 | 158-190 | 55 | MF002384 |
