# Supplementary material for: Estimating seed dispersal distance: A comparison of methods using animal movement and plant genetic data on two primate‐dispersed Neotropical plant species
Source: Ecol Evol. 2019 Jul 25;9(16):8965–77. doi: 10.1002/ece3.5422 (PMC6706201; doi:10.1002/ece3.5422)
Supplement: Supplementary file 6 [file ECE3-9-8965-s006.docx]

**Table S3.** Number of alleles (A_N_), Effective number of allele (A_E_), Observed heterozygosity (H_o_) and expected heterozygosity (H_e_), deviations from HWE are indicated by asterisks (P < 0.05 = *, P < 0.01 = **, P < 0.001 = ***)

| *L. cymosa* [EBQB] (n = 664) | | | | |
| --- | --- | --- | --- | --- |
| Locus | **A** | **A_E_** | **H_o_** | **H_e_** |
| Leo80 | 3 | 1.9 | 0.423 | 0.469** |
| Leo89 | 6 | 1.4 | 0.307 | 0.307 |
| Leo94 | 3 | 1.1 | 0.117 | 0.119 |
| Leo270 | 4 | 1.1 | 0.116 | 0.125** |
| Leo466 | 4 | 1.9 | 0.488 | 0.484 |
| Leo1842 | 4 | 2.1 | 0.495 | 0.514* |
| Leo2254 | 8 | 2.7 | 0.660 | 0.632*** |
| Leo2428 | 4 | 1.5 | 0.317 | 0.349 |
| Leo2433 | 14 | 7.3 | 0.817 | 0.863*** |
| Leo2833 | 6 | 2.6 | 0.562 | 0.609 |
| Leo2853 | 7 | 1.4 | 0.256 | 0.271 |
