# Supplementary material for: Estimating seed dispersal distance: A comparison of methods using animal movement and plant genetic data on two primate‐dispersed Neotropical plant species
Source: Ecol Evol. 2019 Jul 25;9(16):8965–77. doi: 10.1002/ece3.5422 (PMC6706201; doi:10.1002/ece3.5422)
Supplement: Supplementary file 7 [file ECE3-9-8965-s007.docx]

**Supporting Table S4**: Parameter and values used in the simulation of the individual-based model method (IBM). Executable model in Java at https://doi.org/10.5281/zenodo.1471479

| **Name** | **Value** | **Comment** |
| --- | --- | --- |
| **no_cells** | 80 | size of the landscape each direction |
| **scaling** | 25 | size of on cell |
| **sim_time** | 108 | def. time 580 min = 9:40 +/-x hours/day |
| **no_days** | 100 | no of simulated days = 100 days |
| **start_tree_x** | 704 100.34 | 704224.39 |
| **start_tree_y** | 9 517 409.65 | 9517563.63 |
| **tree_file** | all_trees_with_leonia-41.txt | with 41 Leonia trees |
| **start_energy** | 60 | starting energy at day 1 every simulation |
| **energy_level_1** | 80 | if below, feeding |
| **energy_level_2** | 150 | if above, other behaviours |
| **tree_time** | 0.1 | change tree – 5 time steps |
| **tree_time_leonia** | 0.25 | change tree – 2 time steps |
| **feeding** | 8 | gain during feeding per time step |
| **feeding_leonia** | 4 | gain during feeding per time step |
| **running** | −1.6 | loss during running per time step |
| **marking** | −1.7 | loss during marking per time step |
| **foraging** | −0.5 | loss during foraging per time step |
| **resting** | −0.7 | loss during resting per time step |
