# Supplementary material for: Estimating seed dispersal distance: A comparison of methods using animal movement and plant genetic data on two primate‐dispersed Neotropical plant species
Source: Ecol Evol. 2019 Jul 25;9(16):8965–77. doi: 10.1002/ece3.5422 (PMC6706201; doi:10.1002/ece3.5422)
Supplement: Supplementary file 8 [file ECE3-9-8965-s008.docx]

**Supporting Table S5.** Summary of seed dispersal distance estimates in metres and statistics for *Parkia panurensis* (A) and *Leonia cymosa* (B) using different methods (OSD= observed seed dispersal events, GSC = Maternal ID from seed coats, PAS = Parentage analysis, CMG = combination of movement data and gut passage, IBM= individual-based modelling). Mean, Standard deviation (SD), sample number (*n*), Modes, distribution range (5%,, Median, 95%), and results given by bootstrapping with 10,000 permutations are given: Mean (Mean_boot), bias (bias_boot), Standard error (SE_boot).

| ***Parkia panurensis*** | | | |  |  |  |  |  |  |  |  |
| --- | --- | --- | --- | --- | --- | --- | --- | --- | --- | --- | --- |
|  | Mean | SD | *n* | Mode1 | Mode2 | 5% | Median | 95% | Mean_boot | bias_boot | SE_boot |
| **OSD** | 175 | 119 | 269 | 71 | - | 21 | 159 | 404 | 175 | 0 | 7 |
| **GSC** | 158 | 110 | 75 | 65 | 499 | 16 | 134 | 330 | 158 | 0 | 13 |
| **PAS** | 172 | 135 | 38 | 126 | - | 11 | 151 | 467 | 185 | 0 | 14 |
| **CMG** | 188 | 122 | 1647 | 93 | - | 22 | 168 | 419 | 188 | 0 | 3 |
| **IBM** | 201 | 136 | 1180 | 150 | 621 | 27 | 177 | 480 | 201 | 0 | 4 |
|  |  |  |  |  |  |  |  |  |  |  |  |
| ***Leonia cymosa*** | | | |  |  |  |  |  |  |  |  |
|  | Mean | SD | *n* | Mode1 | Mode2 | 5% | Median | 95% | Mean_boot | bias_boot | SE_boot |
| **OSD** | 234 | 111 | 4 | 295 | - | -163 | 258 | 584 | 234 | 0 | 48 |
| **GSC** | 300 | 74 | 9 | 215 | 346 | 97 | 339 | 486 | 300 | 0 | 23 |
| **PAS** | 178 | 201 | 17 | 49 | 612 | -128 | 118 | 639 | 178 | -1 | 47 |
| **CMG** | 318 | 137 | 791 | 240 | 347 | 105 | 315 | 552 | 318 | 0 | 5 |
| **IBM** | 300 | 141 | 194 | 286 | - | 76 | 289 | 557 | 300 | 0 | 10 |
