# Supplementary material for: Estimating seed dispersal distance: A comparison of methods using animal movement and plant genetic data on two primate‐dispersed Neotropical plant species
Source: Ecol Evol. 2019 Jul 25;9(16):8965–77. doi: 10.1002/ece3.5422 (PMC6706201; doi:10.1002/ece3.5422)
Supplement: Supplementary file 9 [file ECE3-9-8965-s009.docx]

# Appendix: Supporting information

## Supporting Information – S1

**Characterization of 11 microsatellites (SSRs) loci for *Leonia cymosa* and their compatibility with 2 congeneric species, *L. glycicarpa* and *L. crassa*.**

Microsatellite development and characterization

Leaf samples of *Leonia cymosa* were collected at Estación Biológica Quebrada Blanco (EBQB) and in the periphery of Allpahuayo-Mishana (AM) in north-eastern Peruvian Amazonia. Samples of *L. glycicarpa* and *L. crassa* were only collected at AM. Location and description of vouchers are described in Table S1.

We stored leaves collected by drying them in silica gel beads (Sigma-Aldrich, St. Louis, MO, USA) or pressed into FTA PlantSaver cards (GE Healthcare, Chicago, IL, USA) and stored seeds collected on saline solution. We extracted DNA from dried leaf tissue and seed coats by homogenizing 100mg of each sample using a Retsch shaking mill (Retsch, Hilden, Germany) and subsequently following the ATMAB-based protocol (Dumolin et al., 1995) with an additional final treatment with 0.5 µl RNase at 37°C for 30 min. For leaf samples stored in FTA PlantSaver cards, we extracted DNA following the company’s protocol and adding a final five min incubation step in TE buffer (TRIS, EDTA) at 95°C for obtaining solute DNA. DNA concentrations were measured using the NanoDrop 3300 (Thermo Fisher Scientific, Waltham, MA, USA). Both methods of storage for leaves yielded identical genotyping results when used on the same samples (unpub. data).

Microsatellites markers were developed by Ecogenics GmbH (Balgach, Switzerland). Size-selected fragments of genomic DNA were enriched for SSR content with magnetic streptavidin beads and biotin-labelled GATA, GTAT, AAAC and AAAG repeat oligonucleotides. The SSR-enriched library was sequenced on an Illumina MiSeq platform using the Nano 2x250 v2 format. After assembly, 3,855 contigs or singlets contained a microsatellite insert with a tetra- or a trinucleotide of at least six repeats or a dinucleotide of at least 10 repeats. Primer design was possible in 171 microsatellite candidates. Out of these, 11 loci were successfully amplified in all 15 screened individuals. For additional testing, we further amplified these 11 loci in 32 individuals of *L. cymosa* on an automatic capillary sequencer (MegaBACE 1000, GE Healthcare, Chicago, IL, USA) with the size standard MegaBACE ET400-R (GE Healthcare, Chicago, IL, USA).

We assembled the primers for the targeted microsatellites in multiplexes according to their annealing temperatures (Table S2).

We performed PCR reactions using the Qiagen Type-it microsatellite PCR kit (Qiagen, Venlo, Netherlands) in 14.6 µl of master solution containing: 2 µl of 10 ng/µl genomic DNA, 8.3 µl 1x Type-it multiplex PCR master mix, 1.2 to 2.0 µl of each 2 µM primer pair solution (based on calibration), and double-distilled water (ddH_2_O). PCR conditions on the thermocycler (T1, Biometra, Goettingen, Germany) were set to 94 °C for 5 min, followed by 34 cycles at 94 °C for 30 s, annealing temperature for 90 s, extension at 72 °C for 30 s and a final extension at 60 °C for 30 min (Table S2). PCR amplification products were again separated on the MegaBACE 1000. Subsequently, we determined the allelic composition of each sample using the MegaBACE Genetic Profiler v. 2, an example of an electropherogram for each primer is given in Figure S1.

***Population genetics analysis***

Effective number of alleles (A_E_), observed heterozygosity (H_O_), expected heterozygosity (H_E_), and deviation from Hardy-Weinberg equilibrium (HWE) were determined using GenAlex v. 6.2 (Peakall and Smouse, 2006) based on 645 genotyped *L. cymosa* individuals from EBQB. Linkage disequilibrium (LD) was tested using GENEPOP version 4.3 (Rousset, 2008). Null alleles were analysed using MICRO-CHECKER v. 2.2.3 (Van Oosterhout et al., 2004).

**Results**

Amplification products of 11 microsatellite primer pairs showed polymorphic bands that could be reliably scored and were used for further analysis. Mean number of alleles of all loci was 5.7 (range 3 – 14) and expected heterozygosity ranged from 0.12 to 0.87 (mean 0.41). Significant deviations from HWE were found in five loci (Table S3). Significant linkage disequilibrium (q-value < 7 x 10^-5^) was detected for Leo89 and Leo466 (Storey and Tibshirani, 2003). Null alleles were detected for Leo2428 (A_N_= 0.053). However, corrected estimated allele frequencies based on null alleles deviated only by 0.02 from the observed frequencies (Table S3).

All microsatellite markers were successfully amplified in the second population of *L. cymosa* and in the congeneric species *L. glycicarpa.* Only seven loci out of 11 were successfully amplified in *L. crassa*. The microsatellite markers were highly variable in the population of *L. cymosa* at EBQB and cross-amplification maintained this high variability. For all sampling sites, fragment length was within the same range as in *L. cymosa* from EBQB.

**References**

Dumolin, S., Demesure, B., & Petit, R. J. J. (1995). Inheritance of chloroplast and mitochondrial genomes in pedunculate oak investigated with an efficient PCR method. Theoretical and Applied Genetics, 91(8), 1253–1256. https://doi.org/10.1007/BF00220937

Gelmi-Candusso, T.A. (2019) Frugivore behavior and plant spatial genetics. Doctoral Dissertation. Georg-August University of Göttingen, Germany.

Van Oosterhout, C., Hutchinson, W. F., Wills, D. P. M., & Shipley, P. (2004). MICRO-CHECKER: Software for identifying and correcting genotyping errors in microsatellite data. Molecular Ecology Notes, 4(3), 535–538. https://doi.org/10.1111/j.1471-8286.2004.00684.x

Peakall, R., & Smouse, P. E. (2006). GENALEX 6: Genetic analysis in Excel. Population genetic software for teaching and research. Molecular Ecology Notes, 6(1), 288–295. https://doi.org/10.1111/j.1471-8286.2005.01155.x

Rousset, F. (2008). genepop’007: a complete re-implementation of the genepop software for Windows and Linux. Molecular Ecology Resources, 8(1), 103–106. https://doi.org/10.1111/j.1471-8286.2007.01931.x

Storey, J. D., & Tibshirani, R. (2003). Statistical significance for genomewide studies. Proceedings of the National Academy of Sciences, 100(16), 9440–9445. <https://doi.org/10.1073/pnas.1530509100>

## Supporting Information – S2

**R function script** for extracting linear travel distances from movement data collected from scan sampling or tracking devices, and for running the CMG method (combination of movement data and gut passage time). Available at https://doi.org/10.5281/zenodo.1470486

####Linear.distances() function

linear.distances <- function (time, year, month, day, xUTM, yUTM){

timeN <- sapply(strsplit(time,":"),

function(x) {

x <- as.numeric(x)

x[1]+x[2]/60#+x[3]/1200

} ) #converts time to decimal (e.g 11:30=11.5, 01:00 = 1.0)

time_interval <- abs(apply(combn(timeN,2), 2, diff))

year_interval <- abs(apply(combn(year,2), 2, diff))

month_interval <- abs(apply(combn(month,2), 2, diff))

day_interval <- abs(apply(combn(day,2), 2, diff))

X_interval <- abs(apply(combn(xUTM,2), 2, diff))

Y_interval <- abs(apply(combn(yUTM,2), 2, diff))

distance_interval <- sqrt((X_interval^2)+(Y_interval^2))

date_interval <- year_interval+month_interval+day_interval

comb <- data.frame (date_interval, distance_interval,time_interval)

daily_linear_travel_paths <- comb[ which(comb$date_interval == 0), ]

daily_linear_travel_paths$date_interval <- NULL

return(daily_linear_travel_paths)

}

#example of input parameteres

time <- as.character(c("11:00", "11:30", "12:00", "12:30", "13:00", "13:30"))

year <- as.numeric(c(2012,2012,2012,2012, 2012))

month <- as.numeric(c(12,12,12,12, 12, 12))

day <- as.numeric(c(14,14,14,14,14,14))

xUTM <- as.numeric(c(704265,704256, 704249, 704146, 704090, 704010))

yUTM <- as.numeric(c(9517640, 9517554, 9517526, 9517567, 9517564, 9517571))

##########to obtain SDD estimates using the CMG method#######

##1. Load data file from csv, time format should be in "%H:%M” or "%H:%M:%S", and date should be separated in columns according to day, month, year.

trial <- read_csv("~/linearmovement_automatization_trial.csv",

locale = locale(date_format = "%Y-%m-%d",

time_format = "%H:%M:%S",tz = "UTC"))

##2. Restrict data to fruiting season

trial[trial$Month %in% c("3","4","5"),]-> trial_FS

##3. Order data chronologically

trial_FS <- trial_FS [order(trial_FS$Year, trial_FS$Month, trial_FS$Day, trial_FS$Time),]

## 4. Determine input parameters for function

as.character(trial_FS$Time) ->time #format "%H:%M" if "%H:%M:%S" then add +x[3]/1200 to function by deleting “#” on line 5.

as.numeric (trial_FS $Year )-> year

as.numeric(trial_FS$Month) -> month

as.numeric(trial_FS$Day) -> day #data points have to be in chronological order

as.numeric(trial_FS$X) -> xUTM

as.numeric(trial_FS$Y) -> yUTM

##5. execute function

linear.distances (time, year, month, day, xUTM, yUTM) -> daily_linear_travel

##6. restrict linear travel paths to those within the gut passage time of a particular plant species or a mean for the animal species.

CMG_SDDestimates <-daily_linear_travel[daily_linear_travel$time %in% c(1,1.5,2),] #e.g. gut passage of 1-2hrs
